# A Computational Approach to Characterize the Protein S-Mer Tyrosine Kinase (PROS1-MERTK) Protein-Protein Interaction Dynamics

**DOI:** 10.1007/s12013-024-01582-5

**Published:** 2024-11-13

**Authors:** Mak B. Djulbegovic, David J. Taylor Gonzalez, Luciano Laratelli, Michael Antonietti, Vladimir N. Uversky, Carol L. Shields, Carol L. Karp

**Affiliations:** 1 Wills Eye Hospital, Thomas Jefferson University, Philadelphia, PA USA; 2 Hamilton Eye Institute, University of Tennessee Science Center, Memphis, TN USA; 3 Atlantico Software, Miami Beach, FL USA; 4 Bascom Palmer Eye Institute, University of Miami, Miami, FL USA; 5 Department of Molecular Medicine and USF Health Byrd Alzheimer’s Research Institute, Morsani College of Medicine, University of South Florida, Tampa, FL 33612 USA; 6 Ocular Oncology Service, Wills Eye Hospital, Thomas Jefferson University, Philadelphia, PA USA

**Keywords:** PROS1, MERTK, AlphaFold, Protein-Protein Interactions, Molecular Dynamics, GROMACS

## Abstract

Protein S (PROS1) has recently been identified as a ligand for the TAM receptor MERTK, influencing immune response and cell survival. The PROS1–MERTK interaction plays a role in cancer progression, promoting immune evasion and metastasis in multiple cancers by fostering a tumor-supportive microenvironment. Despite its importance, limited structural insights into this interaction underscore the need for computational studies to explore their binding dynamics, potentially guiding targeted therapies. In this study, we investigated the PROS1–MERTK interaction using advanced computational analyses to support immunotherapy research. High-resolution structural models from ColabFold, an AlphaFold2 adaptation, provided a baseline structure, allowing us to examine the PROS1–MERTK interface with ChimeraX and map residue interactions through Van der Waals criteria. Molecular dynamics (MD) simulations were conducted in GROMACS over 100 ns to assess stability and conformational changes using RMSD, RMSF, and radius of gyration (*R*g). The PROS1–MERTK interface was predicted to contain a heterogeneous mix of amino acid contacts, with lysine and leucine as frequent participants. MD simulations demonstrated prominent early structural shifts, stabilizing after approximately 50 ns with small conformational shifts occurring as the simulation completed. In addition, there are various regions in each protein that are predicted to have greater conformational fluctuations as compared to others, which may represent attractive areas to target to halt the progression of the interaction. These insights deepen our understanding of the PROS1–MERTK interaction role in immune modulation and tumor progression, unveiling potential targets for cancer immunotherapy.

## Introduction

Protein S (PROS1) is a vitamin K-dependent glycoprotein that plays a crucial role in the anticoagulant pathway by acting as a cofactor for activated protein C, thereby inhibiting coagulation factors Va and VIIIa to prevent excessive thrombosis [1]. Beyond its classical function in hemostasis, PROS1 serves as a ligand for the TAM family of receptor tyrosine kinases—TYRO3, AXL, and MERTK—mediating diverse biological processes such as immune regulation, cell survival, and phagocytosis [2–6].

Tyrosine-protein kinase Mer (MERTK), is a proto-oncogene highly expressed on macrophages, dendritic cells, and various other cell types, including endothelial and epithelial cells [7–11]. Ligand binding to MERTK, including interactions with PROS1, induces autophosphorylation of the intracellular domain, creating docking sites for downstream signaling molecules [12]. This activation leads to the phosphorylation of key proteins, such as MAPK1, MAPK2, and FAK, which regulate critical processes including cell survival, cytoskeletal reorganization, and platelet aggregation [12]. In physiological conditions, the interaction between PROS1 and MERTK is essential for efferocytosis—the clearance of apoptotic cells by phagocytes—which is critical for maintaining tissue homeostasis and preventing chronic inflammation and autoimmunity [13]. Dysregulation of the PROS1-MERTK signaling pathway has been implicated in a spectrum of diseases, including autoimmune disorders, chronic inflammatory conditions, and several types of cancer [2, 3, 12].

In cancer biology, aberrant activation of MERTK by PROS1 contributes to tumor progression by promoting cell survival, proliferation, migration, and immune evasion, and has been implicated in a variety of solid tumors, including lung, breast, and glioblastoma multiforme, where its overexpression correlates with poor prognosis and resistance to apoptosis [14–17]. Elevated levels of PROS1 have also been observed in certain cancers, including lung cancer, where PROS1-mediated signaling enhances tumor cell proliferation, migration, and angiogenesis [3, 18]. In uveal melanoma, a malignancy of the eye, PROS1 is significantly upregulated in class 2 tumors characterized by the loss of BAP1—a tumor suppressor gene [19]. BAP1 deficiency leads to increased PROS1 expression in uveal melanocytes and melanoma cells, resulting in the activation of MERTK on macrophages and their polarization to an immunosuppressive M2 phenotype [19, 20]. This promotes a tumor immune microenvironment (TIME) that facilitates tumor growth and metastasis [19, 21]. The PROS1-MERTK interaction thus represents a promising target for the development of immunotherapies in uveal melanoma and potentially other cancers where this axis is active.

Despite the recognized importance of the PROS1-MERTK interaction in various diseases, detailed structural insights into their binding interface are limited [22]. Advancements in computational biology, particularly in protein structure prediction and modeling, offer powerful tools to elucidate protein-protein interactions at the atomic level [23]. Techniques such as AlphaFold and ColabFold enable high-accuracy predictions of protein structures based solely on amino acid sequences [24–26]. Molecular dynamics (MD) simulations complement these structural predictions by modeling the dynamic behavior of protein complexes under physiological conditions, providing insights into their conformational flexibility and stability [27].

Recent studies have underscored the power of molecular dynamics simulations in providing detailed insights into protein-ligand interactions and complex stability, further validating molecular docking predictions. For instance, simulations have refined our understanding of taste receptor interactions with modified steviosides, while MM-PBSA analyses have elucidated the binding affinities of curcuminoid inhibitors targeting SARS-CoV-2 Nsp15 [28, 29]. Similarly, molecular dynamics has been instrumental in cancer research, demonstrating how point mutations in the p110α subunit of PI3K induce structural flexibility and reduce its interaction with regulatory partners, providing a mechanistic explanation for oncogenic behavior [30]. Additionally, in-silico screening of PLK1 revealed that mutations like W414F lead to structural instability and altered protein-ligand interactions, further linking these changes to cancer pathogenesis [31]. Notably, steered molecular dynamics simulations and umbrella sampling have benchmarked the efficacy of novel 1,2,3-triazole compounds against the SARS-CoV-2 main protease, showing that these compounds exhibit comparable binding free energies and potential mean force to known inhibitors, positioning them as promising antiviral candidates [32]. These examples highlight the versatility and precision of computational approaches in both drug discovery and the understanding of disease mechanisms.

In our previous study, we employed bioinformatics approaches to model the PROS1-MERTK interaction and identified intrinsically disordered protein regions (IDPRs) in both proteins, suggesting that intrinsic disorder may facilitate their interaction [22]. Intrinsically disordered regions lack fixed three-dimensional structures yet are functionally significant, often mediating protein-protein interactions through conformational flexibility [33–35]. Building upon these findings, the present study aims to deepen our understanding of the PROS1-MERTK interaction through additional computational analyses with a particular focus on molecular dynamics. Our objectives are to quantify the interaction interface between PROS1 and MERTK, identify key residues involved in binding, and analyze the structural dynamics of the complex. By elucidating the molecular details of the PROS1-MERTK interaction, we hope to contribute to the development of targeted therapies for diseases where this interaction plays a critical role, especially in the context of TIME.

## Methods

In this study, our methods are aimed at computationally analyzing the dynamics of the PROS1-MERTK complex, a critical interaction implicated in promoting a tumor immune microenvironment in many cancers. Recognizing the potential of this interaction for immunotherapeutic interventions, we have streamlined our approach to exclusively focus on this complex. Our approach allows us to delve deeply into the structural and dynamic properties of the PROS1-MERTK complex, utilizing predictive modeling techniques and molecular dynamics simulations. Each step of our process, from structure generation using the latest iterations of ColabFold to detailed dynamic analyses in GROMACS, is tailored to characterize the unique characteristics of this interaction under physiologically relevant conditions. The subsequent sections outline our refined techniques, emphasizing precision and context-specific accuracy in modeling and simulation, which are crucial for understanding the potential of PROS1-MERTK as a target in cancer immunotherapy.

### Sourcing the PROS1-MERTK Structure for Downstream Analysis

We are utilizing previously generated high-resolution structures of the PROS1-MERTK complex [22]. These structures were computed using ColabFold, an adaptation of AlphaFold2 that leverages Google Colaboratory’s cloud GPUs for enhanced computational speed and accessibility. ColabFold integrates AlphaFold2’s deep learning algorithms to predict protein structures with high precision based on their amino acid sequences. This robust platform ensured that our structural predictions were both accurate and reliable, forming a solid foundation for our current investigations into the dynamics and interactions of the PROS1-MERTK complex. ColabFold predictions for PROS1-MERTK were quantified by predicted local distance difference test (pLDDT). The model confidence is stratified into four categories: very high (pLDDT > 90), confident (90 > pLDDT > 70), low (70 > pLDDT > 50), and very low (pLDDT < 50). The pLDDT score is important for assessing the reliability of predicted protein structures. High pLDDT scores indicate well-modeled regions suitable for detailed analysis. Low pLDDT scores often correspond to intrinsically disordered regions (IDRs), which lack fixed structure but are functionally significant in processes like signaling and regulation. Recognizing these low-confidence areas allows us to consider the potential impact of IDRs on protein behavior.

### Quantitative Analysis of the PROS1-MERTK Interaction Interface

Utilizing the ChimeraX software, we conducted our interface analysis of PROS1-MERTK using the default settings for molecular contact determination [36–38]. We used the default threshold for Van der Waals (VDW) overlap at ≥ −0.40 angstroms (Å). Contacts between atoms separated by four bonds or fewer were not considered, and both intra-residue and intra-molecule contacts were excluded from the detection, ensuring the specificity and relevance of the identified contacts in the PROS1-MERTK protein-protein interaction. We then mapped the frequency of amino acid contacts within the PROS1-MERTK protein-protein interaction. The frequency of amino acid contacts was established by counting the instances each amino acid was involved in contacts that met our VDW criteria (≥ −0.40 Å). We then classified the contacts at each protein interface by their physicochemical properties and structural tendencies. The contacts were categorized into hydrophobic, hydrophilic, ordered, disordered, basic, or acidic.

### Molecular Dynamics of the PROS1-MERTK Protein-Protein Interaction

#### System Set-Up

Transitioning from the analysis of the PROS1-MERTK interface, our focus narrowed to the PROS1-MERTK analyzing the dynamics of the interaction. Our group is interested in the interaction between PROS1 and MERTK as it promotes a tumor immune microenvironment (TIME). Given the significant association of the PROS1-MERTK interaction in many cancers, our investigations centered on this complex to understand its dynamic behavior and stability. The starting structure for our molecular dynamics (MD) simulations was derived from the predicted model generated by ColabFold as previously detailed. This structure provided a high-resolution basis for exploring the atomic-scale movements and interactions within the PROS1-MERTK complex.

Following the model’s creation using ColabFold, the workflow proceeded with a series of steps in the GROMACS environment [39–41]. The initial step involved selecting a force field. We elected to use the all-atom optimized potentials for liquid simulations all-atom (OPLS-AA) force field as it is known for its accurate representation of biomolecular systems [42–44]. After choosing OPLS-AA, we focused on configuring our simulation environment. A part of this setup was defining the dimensions of the simulation box that would house the PROS1-MERTK complex. A cubic box was constructed, ensuring the complex resided at the center with a minimum distance of 1.0 nm from the walls. This consideration is essential as it respects the minimum image convention crucial for periodic boundary conditions in molecular simulations, thereby preventing the protein from interacting with its own replicated images across the boundaries of the box. The size of the box was determined to be large enough to avoid any interactions between the protein and its periodic images, which could lead to erroneous forces within the system.

With our simulation box defined, we next incorporated water molecules using a well-validated water model. The water model chosen was the extended simple point charge (SPC/E) water model. This three-point rigid water model is widely used due to its ability to replicate the physical properties of water with a good balance between accuracy and computational efficiency [45–47]. This model helps ensure that the solvent environment around our complex are both realistic and conducive to meaningful simulation results, allowing us to closely mimic the hydration conditions found in biological systems.

To appropriately represent the ionic environment surrounding the extracellular domain of the PROS1-MERTK complex, ions were added to the simulation to achieve charge neutrality and to emulate the ionic strength typically found in biological fluids. Sodium (Na + ) ions were primarily considered due to their prevalence in extracellular fluids, reflecting the natural conditions under which the interactions of the PROS1-MERTK complex typically occur outside of cellular boundaries. However, potassium (K + ) ions were also included in a parallel setup to assess their influence on the complex. Including K+ ions also serves as a methodological probe, testing the robustness and consistency of the complex’s behavior under varied ionic conditions.

#### Pre-production Energy Minimization and Equilibration

Following the solvation and ion addition to achieve an electroneutral system, the configuration was subjected to energy minimization to ensure that any potential steric clashes or geometric inconsistencies introduced during the solvation and ion placement were resolved. This process, essential for the refinement of the system, involved having GROMCAS iteratively adjust atomic positions to systematically lower the system’s potential energy [48–50]. The energy minimization effectively relaxed the structure, smoothing out the energy landscape by correcting unfavorable overlaps between atoms and thus setting the stage for a physically plausible starting point for the next phase of our computational study, the equilibration of our molecular dynamic environment.

The canonical (NVT) ensemble was employed during the initial equilibration to stabilize the system’s temperature. The NVT ensemble allows for the control of the thermal energy within the system while maintaining the number of particles (N) and volume (V) fixed, reflecting conditions of a closed system with no exchange of matter or volume with the surroundings. This ensemble is particularly useful for ensuring that the system reaches thermal equilibrium at the desired temperature, a prerequisite for realistic dynamic simulations. Following temperature stabilization in the NVT ensemble, we proceeded to pressure equilibration using the isothermal-isobaric (NpT or NPT) ensemble. This ensemble allows the system’s pressure to adjust to a set value by fluctuating the volume while keeping the number of particles (N) and temperature (T) constant.

#### Production Molecular Dynamics

Once we confirmed the equilibration of our system, we proceeded with our production molecular dynamics simulations. These simulations were conducted using GROMACS version 2024.1 on the University of Tennessee’s Infrastructure for Scientific Applications and Advanced Computing (ISAAC). The simulations were run for a duration of 100 ns to comprehensively analyze the dynamic stability and conformational fluctuations of the PROS1-MERTK complex under various ionic conditions. Key metrics such as Root Mean Square Deviation (RMSD), Radius of Gyration (R_g_), and Root Mean Square Fluctuation (RMSF) were calculated for simulations conducted with both sodium (Na + ) and potassium (K + ) ions to elucidate the effects of the ionic environment on the complex’s structure and dynamics.

## Results

### Sourcing PROS1-MERTK Structure for Downstream Analysis

**Fig. 1:**
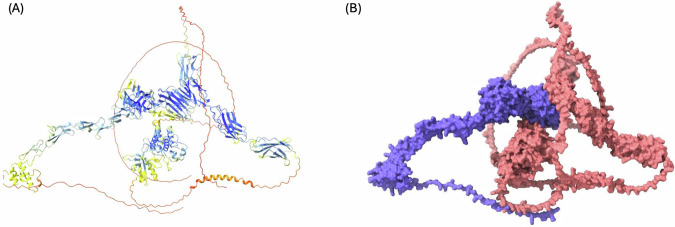
Structural Visualization of PROS1 Protein-Protein Interaction Generated by ColabFold. The average per-residue confidence scores (pLDDT) score for the PROS1-MERTK interaction is 71.12. The structure on the left **A** is color-coded from blue (high confidence) to red (low confidence). For the structure on the right **B** PROS1 is colored in blue and MERTK is salmon colored. These figures are adopted from a prior study by Djulbegovic et al.

Adopted from a prior study by Djulbegovic et al., Fig. 1 presents a visualization of the PROS1-MERTK interaction generated by ColabFold [22]. For the protein-protein interaction, the left panel depicts the traditional AlphaFold confidence coloring, while the right panel utilizes a distinct color-coding scheme to highlight interaction points: PROS1 is depicted in blue, and its binding partner MERTK is shown in a salmon color. The PROS1-MERTK complex, with an average pLDDT of 71.12 (Fig. 1), reveals moderate confidence throughout PROS1’s structure, which appears consistent in shape with a slightly undulating tail region.

### Quantitative Analysis of Protein-Protein Interaction Interfaces

Quantitative analysis of the PROS1-MERTK interface demonstrates a broad spectrum of amino acids involved with the interaction, suggesting a heterogeneous interface between these two proteins (Table 1). The sum of residue-residue contacts is defined as the total number of contact points at the interaction face predicted by ColabFold, which is calculated by ChimeraX. The PROS1-MERTK complex comprises 13,005 atoms and 1675 residues, featuring 334 unique residue-residue contacts.

**Table 1:** Analysis of PROS1 in Protein-Protein Interactions

Protein-protein interaction	Number of atoms	Number of Residues	Contacts^a^
PROS1-MERTK	13,005	1675	334

^a^Contacts represent the count of unique residue-residue contacts between PROS1 and the interaction partner

Summary of the structural components and interface contact of PROS1 when interacting with different protein partners. Each entry details the atom count, residue count, and contact points specific to the interaction.

**Fig. 2:**
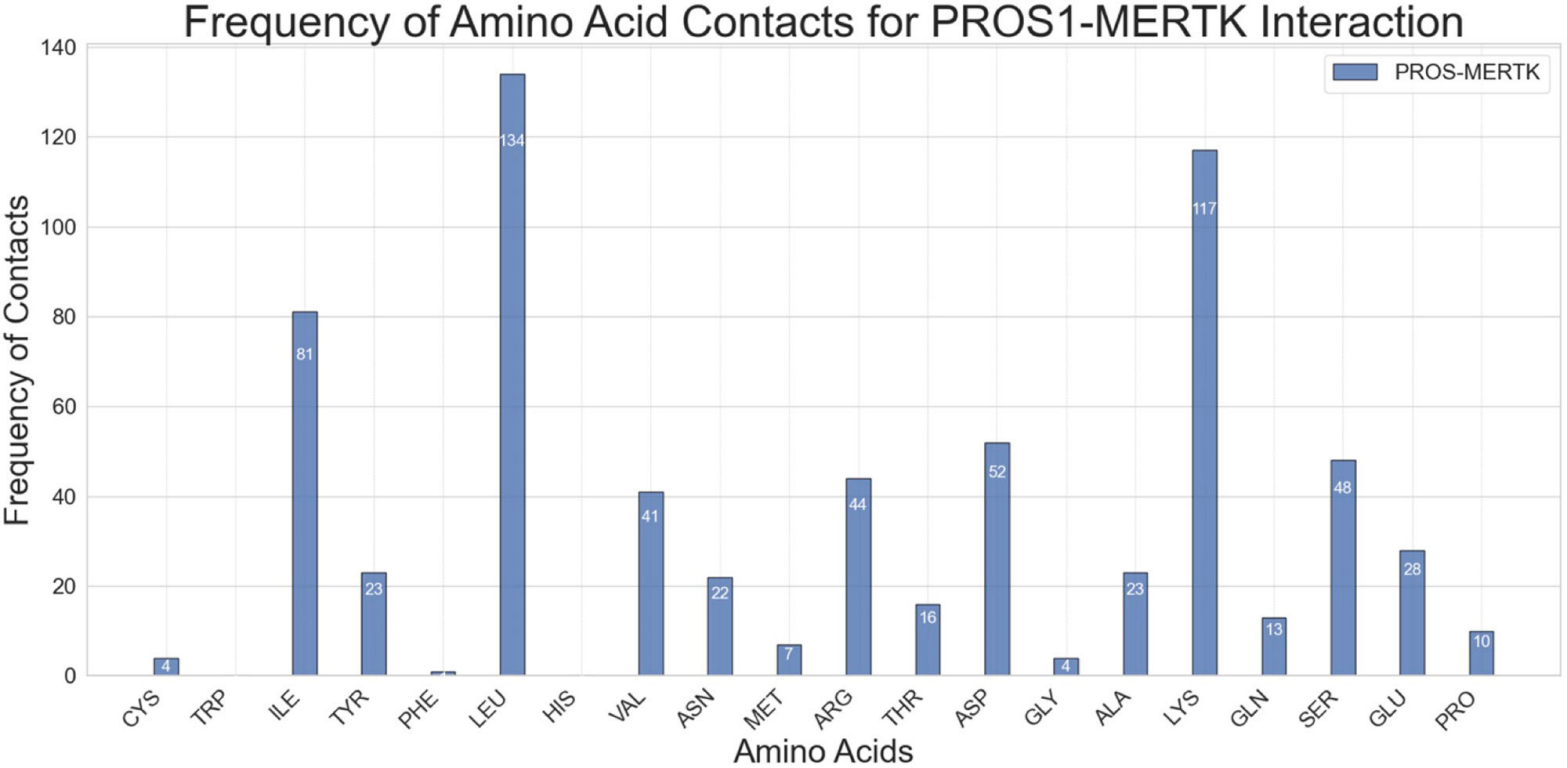
Quantification of Amino Acid Contacts for the PROS1-MERTK protein-protein interaction. The frequency of amino acid contacts across the PROS1-MERTK interface face

Next, we mapped the frequency of amino acid contacts involved in the PROS1-MERTK protein-protein interaction (Fig. 2). Lysine (LYS) had 117 contacts, second only to Leucine (LEU), the most contacted amino acid, with 134 instances. Notably, Arginine (ARG) and Isoleucine (ILE) also contribute substantially, with 44 and 81 contacts respectively, highlighting their potential roles in the binding interface

Further quantitative analysis of the PROS1-MERTK suggests a multifaceted landscape of molecular contacts (Table 2). For the PROS1-MERTK complex, out of a total of 334 contacts, 37.13% were hydrophobic, and 62.87% were hydrophilic in nature. The interface also leaned towards disorder, with 65.87% of contacts classified as disordered, compared to 34.13% as ordered. Basic amino acids played a prominent role, making up 28.14% of the contacts, while acidic amino acids were less prevalent, accounting for 15.87%. Salt bridges were observed in 20.36% of the interactions.

**Table 2:** Composition of Residues at the interface of PROS1 and its protein binding partners

Protein-Protein Interaction	Total Contacts	Hydrophobic	Hydrophilic	Ordered	Disordered	Basic	Acidic	Salt Bridges
PROS1-MERTK	334	37.13%	62.87%	34.13%	65.87%	28.14%	15.87%	20.36%

Interface characteristics between PROS1 and its binding partners MERTK, F5, GGCX, C4B, and TYRO3. The ‘Hydrophobic’ and ‘Hydrophilic’ columns indicate the percentage of interactions involving hydrophobic/nonpolar or hydrophilic/polar amino acids, respectively. ‘Ordered’ and ‘Disordered’ columns show the distribution of amino acids that are promoting structure or lack of structure. The ‘Basic’ and ‘Acidic’ columns provide the percentage of interactions involving amino acids with basic or acidic properties, which are relevant for salt bridge formation. The ‘No. Salt Bridges’ column counts the predicted number of salt bridges identified, and the ‘% Salt Bridges’ column indicates the proportion of salt bridges relative to the total interactions

### Molecular Dynamics of the PROS1-MERTK Protein-Protein Interaction

#### System Set-Up

In our investigation of the dynamic behavior and stability of a protein-protein interaction complex using molecular dynamics (MD) simulations, we employed the OPLS-AA force field across two distinct setups to maintain a consistent representation of biomolecular systems. Each simulation was conducted in a cubic box populated with 809,113 water molecules modeled by the extended simple point charge (SPC/E) water model to closely mimic biological hydration conditions. To explore the influence of ionic environments on the complex, we introduced 40 cations ions into each simulation as the system had a −40 charge before neutralization, employing sodium (Na) ions in one and potassium (K) ions in the other, achieving charge neutrality and emulating the ionic strength typical of biological systems (net charge 0). This setup allowed for a direct comparison between the two ionic conditions, providing insight into their respective impacts on the complex’s behavior and stability.

#### Pre-production Energy Minimization and Equilibration

Our computational analyses of the PROS1-MERTK complex, under varying ionic conditions, successfully progressed through stages of energy minimization, temperature equilibration, and density equilibration, ensuring a physiologically relevant setup for molecular dynamics simulations. Energy minimization confirmed the effective reduction of potential energy in both sodium (Na) and potassium (K) environments, as depicted in Supplemental Fig. S1. This stage resolved any steric clashes and geometric inconsistencies, providing a refined system for subsequent equilibration phases.

Temperature stabilization was achieved within the canonical (NVT) ensemble, demonstrating consistent temperature control over 100 picoseconds in both ionic conditions, which is essential for accurate dynamic simulations (Supplemental Fig. S2). Further equilibration under the isothermal-isobaric (NPT) ensemble allowed our system to adjust to a set pressure, reaching stable conditions indicative of the simulated environment’s density. This process is visually represented in Supplemental Figs. S3 and S4, showing that both Na and K environments achieved temperature stability and density near the target of 1 atm and 1000 kg/m^3^, respectively.

#### Production Molecular Dynamics

**Fig. 3:**
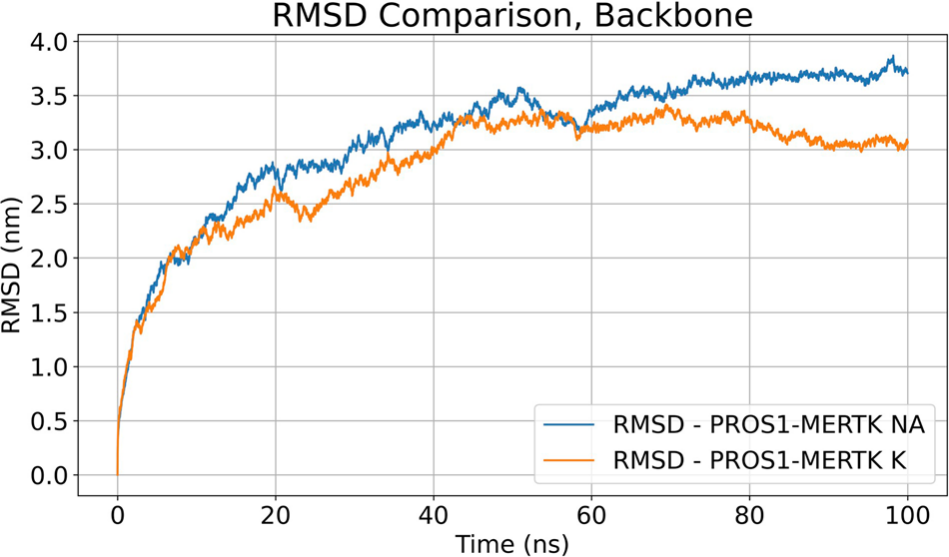
Root Mean Square Deviation (RMSD) comparison of PROS1-MERTK with NA and K. RMSD of the protein backbone over a simulation time of 100 ns

In our molecular dynamics simulations, the root-mean-square deviation (RMSD) profiles for the backbone of the PROS1-MERTK complex in both sodium (Na) and potassium (K) environments were plotted over a simulation time of 100 ns. The RMSD serves as a measure of structural deviation from the initial protein configuration over time. According to Fig. 3, the RMSD trajectories for both ion types exhibit a similar pattern of increase, suggesting that the complex undergoes similar conformational adjustments in each environment. There is an initial rapid rise in RMSD for both Na and K environments to approximately 2.25 nm by 10 ns, indicating significant initial conformational adjustments. After this initial phase, between 10 ns and 50 ns, the RMSD values for both systems increase gradually, with PROS1-MERTK NA consistently maintaining slightly higher values than PROS1-MERTK K. PROS1-MERTK NA fluctuates between 2.25 and 3.50 nm, while PROS1-MERTK K has similar range of RMSD with slightly lower values between these two-time points. Late time progression from 50 ns to 100 ns, both systems exhibit a more stabilized RMSD profile. PROS1-MERTK NA fluctuates between 3.25 and 3.75 nm, while PROS1-MERTK K remains between 3.00 and 3.25 nm. Both systems show small fluctuations, indicating some structural changes but no drastic deviations after 50 ns. At 100 ns, the final RMSD for PROS1-MERTK NA is approximately 3.75 nm, while PROS1-MERTK K finishes near 3.0 nm.

**Fig. 4:**
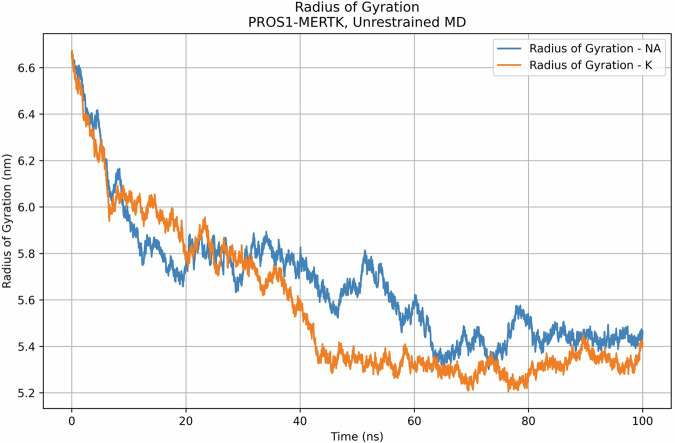
Radius of gyration (R) of PROS1-MERTK with NA and K. Rg over a 100 ns molecular dynamics simulation. This is comparing the compactness of the protein structure over time under two ionic conditions

The molecular dynamics simulation provided additional quantitative insights into the structural dynamics of the PROS1-MERTK complex over a 100 ns period under both sodium Na and K ionic conditions, as measured by the radius of gyration (R_g_) (Fig. 4). The R_g_, which is indicative of the protein’s compactness, showed distinct patterns for each ion type. At the beginning of the simulation, both systems begin with a R_g_ near 6.6 nm. PROS1-MERTK NA and PROS1-MERTK K display nearly identical values during the first few nanoseconds. From 0 to 10 ns, both systems experience a rapid decrease in the R_g_ within the first 10 ns, dropping to approximately 6.0 nm. As the simulation progressed from 10 ns to 50 ns, the two systems diverged in behavior. PROS1-MERTK NA R_g_ values stabilize and appear to be in a local minimum as they fluctuate between 6.0 and 5.8 nm, eventually showing further collapse of the complex when the R_g_ is 5.6 nm. During the 10 ns to 50 ns interval, PROS1-MERTK K has a gradual decline of R_g_ as it collapses from 6.0 to below 5.4 nm. During the final stages of the simulation (i.e., 50 ns to 100 ns), both systems exhibit more stable behavior and appear to have similar levels of compactness at the simulation end time. PROS1-MERTK NA R_g_ was initially higher at 5.8 nm before slowly lowering to approximately 5.4 nm at 65 ns. PROS1-MERTK K maintained an R_g_ around 5.4 nm, showing minimal deviation in the last 55 ns of the simulation At 100 ns, the final R_g_ for both PROS1-MERTK ionic systems was approximately 5.4 nm.

**Fig. 5:**
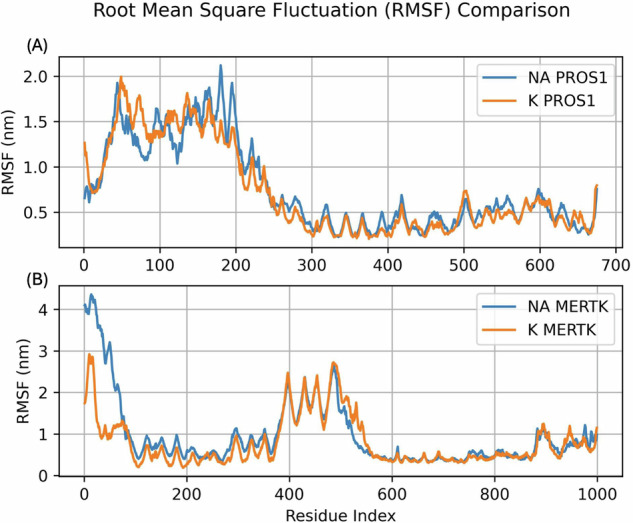
Root Mean Square Fluctuation (RMSF) comparison for **A** PROS1 and **B** MERTK protein chains in NA and K ionic conditions. RMS of each atom within the protein complex across the simulation of 100 ns, with the atom index on the x-axis corresponding to the sequential residue number of each protein chain

The root mean square fluctuation (RMSF) for the PROS1-MERTK complex, comparing the flexibility of the complex in Na and K ionic environments, is shown in Fig. 5. The RMSF values span across the protein’s residues, providing a detailed map of atomic deviations throughout the simulation. Figure 5A demonstrates the RMSF for both ionic environments (i.e., Na vs. K) show similar fluctuation patterns across the residues of PROS1. Fluctuations are highest in the amino acid residues 50 to 200 (1.0 nm < RMSF < 2.0), indicating greater flexibility or movement in the N-terminal region as compared to the rest of the protein. From approximately residue 250 to the C-terminus, both ionic conditions maintain relatively lower RMSF values (RMSF < 0.5 nm), indicating regions of greater structural stability within PROS1. Figure 5B demonstrates similar agreement between both ionic conditions across the residues of MERTK. The first 200 residues exhibit very high RMSF values, particularly in the Na environment, which briefly surpasses 4.0 nm. The K environment demonstrates similar trend in RMSF in the first 100 residues, peaking at nearly 3.0 nm. These findings suggest a highly flexible N-terminal domain. Another region that demonstrates elements of structural flexibility is between residues 375 to 575, where RSMF fluctuates between 1.5 nm to 2.5 nm. Lastly, there are lower levels of fluctuation between residues 100 and 375 and between residues 550 to the C-terminus as both ionic environments show agreement with fairly low RMSF, indicating lower flexibility as compared to the remainder of the protein.

## Discussion

Our study advances the understanding of the PROS1-MERTK interaction interface and structural dynamics, building upon the foundational insights from our group’s prior work. Interface analysis of the protein-protein interactions provided quantitative metrics that suggest a heterogeneous interface consisting of nearly all amino acid types. Notably, the initial binding of PROS1-MERTK contains many salt bridges (20.36% of the total binding interface). Once we established that there is binding specificity by PROS1 with MERTK, our molecular dynamics simulations showed the dynamic behavior of the PROS1-MERTK complex. These simulations consistently highlighted the potential flexibility and responsiveness of the complex, as evidenced by changes in RMSD, the R_g_, and the RMSF profiles. Specifically, the RMSD profiles revealed early and pronounced conformational shifts, with a peak adjustment greater than 3.50 nm in both ionic environments, illustrating the complex’s robust adaptability under varying conditions. Similarly, the radius of gyration (R_g_) underscored dynamic structural changes, notably decreasing from 6.6 nm to 5.4 nm, which reflects a potential collapse of the PROS1-MERTK protein-protein interaction over time. Lastly, the RMSF profiles predicted distinct regions of high flexibility, particularly within the N-terminal domains of both proteins. These pronounced deviations highlight critical areas of the protein complex that might be key targets for therapeutic intervention or further biochemical investigation. These dynamic insights into the PROS1-MERTK complex, highlighted by significant structural adaptability and the presence of specific binding interfaces, offer promising therapeutic avenues for cancers where MERTK signaling is deregulated. Targeting the flexibility and specific interaction points of this complex could lead to the development of novel inhibitors that disrupt these key pathways in cancers such as uveal melanoma, lung cancer, breast cancer, and glioblastoma multiforme [14–19].

The high occurrence of salt bridges within the PROS1-MERTK interaction, constituting over a fifth of the total interactions between the two proteins, emphasizes the strong and specific nature of this PPI. Salt bridges, which form through electrostatic interactions between oppositely charged residues, are possibly critical in fortifying the interaction’s stability and affinity [51, 52]. These electrostatic interactions may stabilize the complex and may also play a pivotal role in the specificity of binding, potentially influencing the biological outcomes of PROS1-MERTK signaling. The prevalence of salt bridges in the PROS1-MERTK interface might enhance the binding affinity and stability of this interaction, potentially amplifying these immunosuppressive signals that allow for the unchecked proliferation of malignant cells. Furthermore, the sensitivity of salt bridges to changes in the cellular microenvironment, such as pH and ionic strength, might influence the PROS1-MERTK interaction under different physiological or pathological conditions. In the acidic microenvironment often found within tumors, alterations in salt bridge interactions could modulate the strength of PROS1 binding to MERTK, thereby affecting the extent of immunosuppression. Understanding the functional implications of these salt bridges may provide valuable insights into the molecular mechanisms by which PROS1-MERTK interactions contribute to cancer progression. This knowledge could inform the development of therapeutic strategies aimed at disrupting these specific interactions to modulate MERTK signaling. For instance, targeting the salt bridges at the binding interface might be a potential approach to inhibit the PROS1-MERTK interaction, thereby alleviating immunosuppression and enhancing anti-tumor immune responses.

Moreover, the molecular dynamics simulations provide a dynamic picture of the PROS1-MERTK complex. The presence of a plateau in the RMSD trajectory implies that the complex has reached a stable conformational state within the 100 ns simulation timeframe. In Fig. 3, we observed notably large RMSD values. These larger RMSD values are primarily due to the extended timeframe of our simulations and the substantial size of the PROS1-MERTK complex. One structural feature significantly influencing these values is the extensive ‘tail’ of MERTK that wraps around PROS1. This wrapping introduces substantial conformational flexibility and dynamism within the complex, contributing to the observed large fluctuations. Such behavior is expected and essential to capture, as it reflects the complex’s physiological motion and interactions, providing a realistic insight into its dynamic structural integrity and function. This highlights the importance of considering the geometric and interactive complexity of protein complexes when evaluating RMSD in molecular dynamics simulations. Additionally, the R_g_ data settles into a plateau, indicating that the final PROS1-MERTK complex achieves a stable, compact conformation as compared to its initial structure. These findings demonstrate a behavior of the complex that aligns with contemporary views of proteins as flexible entities, with their functional states modulated by ongoing structural adaptations [53–56]. Such flexibility might be crucial for the complex’s interactions with other cellular components and signals, affirming the need for proteins to remain dynamic to meet various cellular demands.

Our group previously demonstrated that the PROS1 and MERTK proteins have intrinsic protein disorder [22]. Incorporating the consideration of intrinsically disordered proteins into the molecular dynamic simulations of the PROS1-MERTK complex enriches our understanding of the structural dynamics at play. The RMSD profiles over a 100-ns period suggest an equilibrium in the complex’s conformation with subtle degrees of fluctuation at the end of simulation time. Such ongoing conformational changes may be reflective of intrinsically disordered regions within PROS1, which are known for their dynamic nature and ability to engage in versatile interactions. The relative peaks observed in the R_g_ data suggest the complex explores multiple conformational states, likely due to the influence of disordered regions. Intrinsically disordered proteins (IDPs) are known for their ability to transition between conformations, allowing the complex to maintain functional flexibility while adapting to various environmental cues. These shifts in R_g_ reflect the capacity of PROS1’s intrinsic protein disorder to explore diverse conformational landscapes, which may be important for facilitating the interaction of PROS1 and MERTK overtime. The observed RMSF along the protein chain, particularly the pronounced peaks at terminal regions and specific internal segments, correspond to these disordered regions of the protein. The RMSF data highlights the complex’s inherent flexibility, potentially arising from disordered domains that are conducive to the complex’s functional adaptations. Taken together with the findings presented in our previous study on PROS1, intrinsically disordered protein regions may contribute to the phenomena we have observed in this computational study. These observations are also consistent with our previous studies that demonstrate that intrinsically disordered proteins and disordered protein regions may have a role in oncologic diseases [22, 57–59].

In continuing our efforts to characterize the PROS1-MERTK interaction, subsequent studies may focus on back-engineering the interaction interface. Bioinformatics tools could be used to map out the interaction face of PROS1-MERTK. Once we identify the exact binding zones on both proteins, we will use these as docking sites in molecular docking simulations [60–62]. These simulations would be helpful for the prediction of molecules that may inhibit the PROS1-MERTK interaction. We also aim to analyze the disorder-to-order Order Transition (DOT) regions identified by our study [22]. Specifically, the regions that include cysteine residues at positions 58 and 63 (DOT region #1) and the cluster of 22 cysteines within residues 103 to 261 (DOT region #2) appear to be pivotal for the binding dynamics. Technical examination of the RMSF data indicates a substantial peak within the residue index that correlates to the N-terminus of PROS1, where these DOT regions reside. This peak is indicative of heightened atomic mobility, which aligns with the potential for these cysteine-rich domains to influence the complex’s functional conformation and stability. Targeting these cysteine-rich, disordered-to-ordered transition regions could offer a novel approach to developing immunotherapeutic agents that disrupt the PROS1-MERTK interaction. For example, small molecules or antibodies could be designed to bind to these flexible DOT regions, preventing MERTK signaling and potentially enhancing anti-tumor immune responses in oncologic therapies.

Lastly, our choice to use a ColabFold structure for the PROS1-MERTK protein complex in this study stems from the fact that we previously generated this structure. AlphaFold, developed by DeepMind, is widely recognized for its exceptional accuracy in predicting protein structures, as demonstrated in the CASP competition [24, 25]. ColabFold, which builds on AlphaFold’s algorithms, offers a faster and more accessible platform, making it a valuable tool for generating high-quality protein structures [26]. Alternative protein prediction tools like ESMFold and RoseTTAFold are emerging with promising capabilities [63, 64]. Future studies could focus on generating new structures using these alternative methods and comparing the dynamics of the complex over time, potentially revealing differences in protein behavior that could provide novel insights into how these proteins interact and function under varying conditions.

## Limitations

In this study, we focused on elucidating the dynamic behavior and structural changes of the PROS1-MERTK complex over a 100 ns simulation period. While our analyses primarily centered on RMSD and RMSF to assess the complex’s stability and flexibility, we recognize the potential value of integrating thermodynamic measurements to quantify binding strength and mechanisms. However, our objective was to observe and describe the dynamic interaction process rather than quantifying binding affinity or determining detailed binding mechanisms. This decision was guided by the intent to deepen the understanding of the dynamic interplay within the PROS1-MERTK complex under various conditions. Future studies could build on our findings by incorporating calculations of binding energies or Gibbs free energy to offer comprehensive insights into the thermodynamics of these interactions, which could further elucidate the biological implications of these molecular interactions.

Furthermore, we prioritized investigating the overall structural stability and large-scale conformational dynamics, focusing on phenomena that influence the macroscopic behavior of the complex. Consequently, we did not specifically adjust the protonation states of amino acids, as these finer details were deemed less critical to our primary objectives. This decision was informed by preliminary evaluations suggesting that the key aspects of the complex’s function and interaction dynamics could be robustly captured without this level of granularity. By simplifying the computational requirements, we were able to efficiently explore and elucidate the complex’s structural responses and mechanical properties under various conditions, providing valuable insights into its biological behavior.

While our study has provided substantial insights into the PROS1-MERTK protein-protein interaction, it is not without its limitations. While the SPC/E water model employed in our simulations is widely recognized for its ability to replicate the thermodynamic properties of bulk water, it is acknowledged that the choice of water model can have substantial effects on the behavior of biological macromolecules in simulations. Therefore, future studies may benefit from evaluating the influence of alternative water models [45–47]. Additionally, the exclusive use of the OPLS force field in our simulations necessitates comparative studies with alternative force fields, such as Chemistry at HARvard Molecular Mechanics (CHARMM) [65] and those specifically optimized for intrinsically disordered proteins such as ff14IDPSFF [66], environmental specific precise force field (ESFF1) [67], and Amber ff03 force field (ff03CMAP) [68]. Expanding the scope of our simulations to include a range of water models and molecular force fields could enrich our understanding of protein dynamics and interactions. Such an approach may allow for the exploration of how different computational representations of intermolecular forces and solvent interactions influence the behavior of biological macromolecules. These variations are critical to consider, as they could lead to divergent outcomes in the simulation results, potentially offering alternative interpretations of protein structure, dynamics, and function.

Lastly, during the course of our study, AlphaFold3, a new model capable of predicting the structures and interactions of proteins, DNA, RNA, and small molecules like ligands, was released in a beta phase, a significant advancement with improved accuracy in modeling complex biomolecular interactions, surpassing AlphaFold2 in predictive capabilities [69]. Additionally, the recent development of AlphaProteo, an AI system engineered to create novel, high-affinity protein binders, marks another major breakthrough in structural biology and protein engineering [70]. This advancement has the potential to complement AlphaFold3’s capabilities in drug discovery and the study of protein-protein interactions, promising to streamline the identification of therapeutic targets and accelerate drug development. Given that the AlphaFold3 server is still in its early stages and AlphaProteo is not available, we opted to continue with the well-established AlphaFold2 multimer through ColabFold to generate high-resolution structural models for the PROS1-MERTK complex. In the future, the improved capabilities of AlphaFold3, especially in handling complex biomolecular systems, along with the innovative protein-binder design potential of AlphaProteo, could significantly advance research into protein-ligand interactions. These tools are likely to broaden the scope of structural biology, enabling more accurate predictions and facilitating the development of novel therapeutic strategies across various fields of biomedical research.

Our project provides an illustrative example of how structural models derived from primary amino acid sequences can be paired with molecular dynamics simulations to examine protein dynamics. This approach is in line with existing research methods in the field and contributes to the ongoing validation of computational techniques in predicting protein interactions. While our findings represent a cautious step forward, we recognize the necessity of continual investigation through computational means to further probe PPIs. Notably, integrating our computational work with empirical data from experimentalists will be essential. Importantly, the direct experimental validation of these specific protein-protein contact frequencies and their functional roles remains to be conducted. The evidence confirming the existence of this interaction, however, is supported by existing literature which underscores the biological significance of the PROS1-MERTK pathway in processes. The collaborative efforts between computational biologists and experimentalists are expected to refine our understanding of this PPI, which may pave the way for breakthroughs in the development of targeted immunotherapies for conditions such as uveal melanoma and other cancers.

## Conclusion

In conclusion, our study provides critical insights into the structural and dynamic nature of the PROS1-MERTK interaction, highlighting the complex interplay between protein flexibility, binding specificity, and potential therapeutic relevance. By combining computational modeling with molecular dynamics simulations, we have mapped the interaction landscape and identified regions of functional importance, including salt bridges and intrinsically disordered domains. These findings enhance our understanding of how the PROS1-MERTK complex modulates key biological processes, such as immunosuppression to promote TIME and open new avenues for targeting this interaction in therapeutic contexts. Importantly, the identification of DOT regions, which correspond with areas of high RMSF, presents an opportunity for drug discovery, positioning this complex as a strategic target for the development of novel immunotherapies aimed at disrupting tumor-promoting pathways.

## Supplementary information

Supplementary Information

## Data Availability

Data is provided within the manuscript or supplementary information files.

## Abbreviations

PROS1: (Protein S)
TIME: (tumor immune microenvironment)
RMSD: (root mean squared deviation)
R_g_: (radius of gyration)
RMSF: (root mean square fluctuation)
TYRO3: (Tyrosine-protein kinase receptor TYRO3)
AXL: (Tyrosine-protein kinase receptor UFO)
MERTK: (Tyrosine-protein kinase Mer)
IDPRs: (intrinsically disordered protein regions)
UCSF: (University of California San Francisco)
pLDDT: (predicted local Distance Difference Test)
MSA: (Multiple Sequence Alignment)
VDW: (Van der Waals)
MD: (molecular dynamics)
OPLS-AA: (optimized potentials for liquid simulations all-atom)
SPC/E: (simple point charge)
Na+: (sodium)
K+: (potassium)
N: (number of particles)
V: (volume)
T: (temperature)
ILE: (isoleucine)
LEU: (leucine)
ARG: (arginine)
LYS: (lysine)
TRP: (tryptophan)
PHE: (phenylalanine)
ASN: (asparagine)
GLN: (Glutamine)
GLU: (Glutamic acid)
CYS: (cysteine)
VAL: (valine)
THR: (threonine)
PPIs: (protein-protein interactions)
DOT: (disorder-to-order Order Transition)
CHARMM: (Chemistry at HARvard Molecular Mechanics)
ESFF1: (environmental specific precise force field)
ff03CMAP: (Amber ff03 force field)
