# Supplementary material for: A Computational Approach to Characterize the Protein S-Mer Tyrosine Kinase (PROS1-MERTK) Protein-Protein Interaction Dynamics
Source: Cell Biochem Biophys. 2024 Nov 13;83(2):1743–55. doi: 10.1007/s12013-024-01582-5 (PMC12089150; doi:10.1007/s12013-024-01582-5)
Supplement: Supplementary file 1 — Supplementary Information [file 12013_2024_1582_MOESM1_ESM.docx]

**Supplementary Materials**

A Computational Approach to Characterize the Protein S-Mer Tyrosine Kinase (PROS1-MERTK) Protein-Protein Interaction Dynamics

**Authors:** Mak B. Djulbegovic MD MSc^1^, David J. Taylor Gonzalez MD^2^, Luciano Laratelli MSc^3^, Michael Antonietti BS^4^, Vladimir N. Uversky PhD DSc^5^, Carol L. Shields MD^1,6^, Carol L. Karp MD^4^

**Affiliations:** ^1^Wills Eye Hospital, Thomas Jefferson University, Philadelphia, PA; ^2^ Hamilton Eye Institute, University of Tennessee Science Center, Memphis, TN; ^3^Atlantico Software, Miami Beach, Florida; ^4^Bascom Palmer Eye Institute, University of Miami, Miami, FL; ^5^Department of Molecular Medicine and USF Health Byrd Alzheimer’s Research Institute, Morsani College of Medicine, University of South Florida, Tampa, Florida 33612, USA; ^6^Ocular Oncology Service, Wills Eye Hospital, Thomas Jefferson University, Philadelphia, PA, USA.

**Correspondence:**

Carol L. Karp, MD

Address: 900 NW 17^th^ Street, Miami, FL 33136

Telephone: 305-326-6156

Email: [ckarp@med.miami.edu](mailto:ckarp@med.miami.edu)

**Table of Contents of Supplementary Materials**

- Supplemental Figure S1. Energy Minimization of PROS1-MERTK in NA and K Environments.
- Supplemental Figure S2. Temperature Equilibration of PROS1-MERTK in NA and K Environments.
- Supplemental Figure S3. Equilibration of PROS1-MERTK in NA and K Conditions.
- Supplemental Figure S4. Density Equilibration of PROS1-MERTK in NA and K Conditions.

**Supplemental Figures**


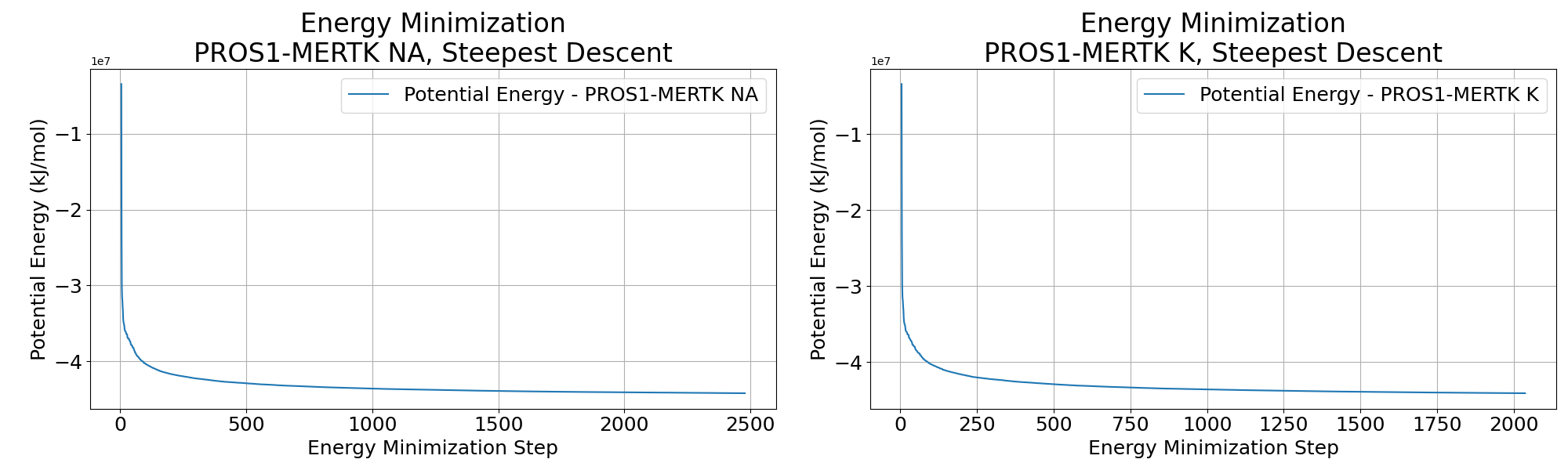


Supplemental Figure S1. Energy minimization of PROS1-MERTK in NA and K environments. The two graphs show the potential energy reduction during the steepest descent energy minimization process for the protein in sodium (NA) and potassium (K) respectively.


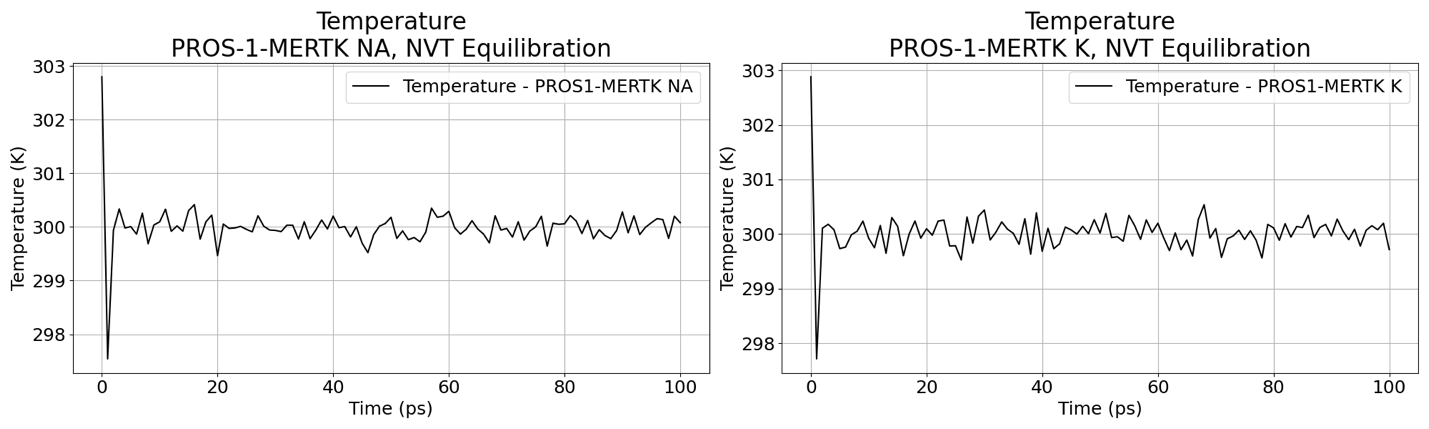


Supplemental Figure S2. Temperature equilibration of PROS1-MERTK in NA and K environments. The left panel shows the temperature stability of PROS1-MERTK during the NVT (constant number of particles, volume, and temperature) equilibration process in a sodium (NA) environment. The right panel depicts the same for a potassium (K) environment, demonstrating how the protein system maintains a consistent temperature over 100 picoseconds.


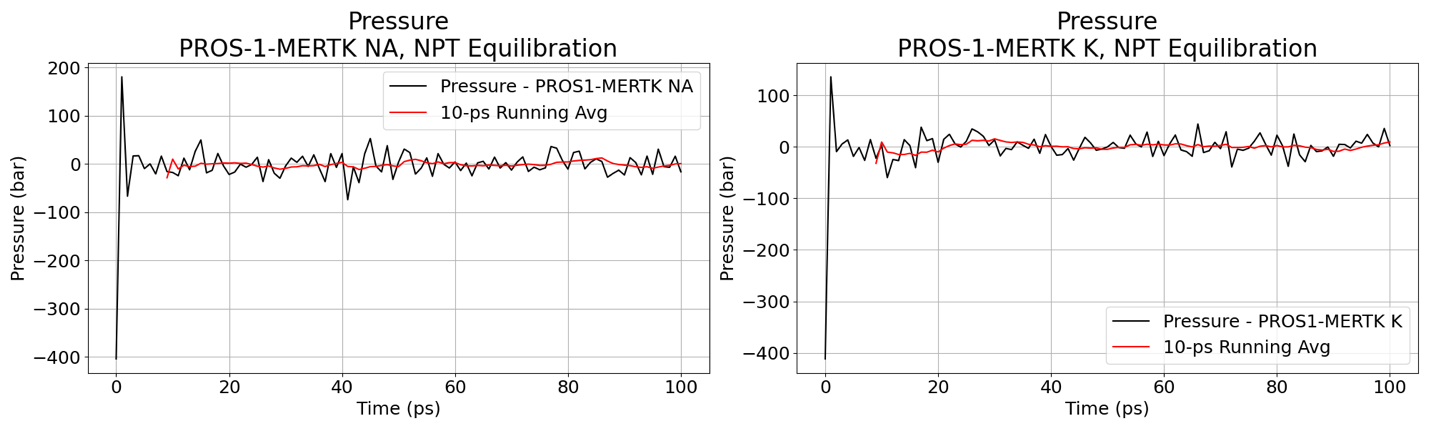


Supplemental Figure S3. Equilibration of PROS1-MERTK in NA and K conditions. The left panel shows the temperature stability of the protein in a sodium environment during NPT or NpT equilibration, while the right panel displays the temperature stability in a potassium environment. Both panels demonstrate the protein reaching a stable pressure around the target of 1 atm under both ionic conditions.


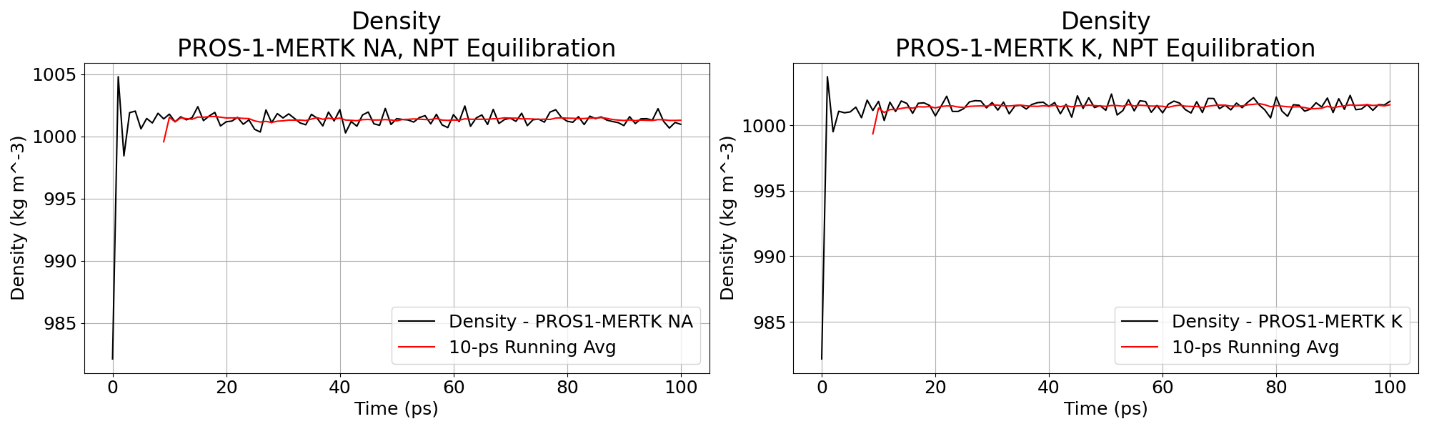


Supplemental Figure S4. Density equilibration of PROS1-MERTK in NA and K conditions. The left panel illustrates the density stability of the protein in a sodium environment during NPT equilibration, while the right panel shows the density stability in a potassium environment. The 10-picosecond running average suggests that both systems reach a stable density near the 1000 kg/m^3 mark.
